# Online teaching and copyright from the European Union perspective in COVID times

**DOI:** 10.1007/s12689-022-00096-8

**Published:** 2022-06-13

**Authors:** Sebastián López Maza

**Affiliations:** Autonomous University of Madrid, Madrid, Spain

**Keywords:** Copyright, Covid-19, Online teaching, Quotation, Private copy, Illustration for teaching

## Abstract

Teaching may involve the use of copyrighted works. It is very important that teachers and professors know two things: (1) what kind of contents they can use in their classes; (2) what are the necessary requirements for using them to avoid infringements of copyright. In this time of pandemic, where online teaching is a big focus in universities, high schools and schools, this knowledge is essential because, despite the alarm generated by COVID-19, copyright is still fully in force. However, copyright is not absolute, as the legislator has provided some limitations or exceptions to these rights. In these cases, it is not necessary to request the consent of the authors and other copyright holders, but the works and subject matters can be used provided that certain requirements are met. In the field of education and teaching, three limitations are applied in particular: (a) private copying; (b) quotation; (c) illustration for teaching.

## Introduction

There is no doubt that the pandemic situation we are living now has changed many of the tasks that we were developing previously. One of them is teaching. Schools, high schools and universities have been forced to change their face-to-face teaching model for an online teaching one. The effort developed by the teachers and professors has been very huge. In this adaptation, it is possible that professors and teachers may have used copyrighted content, either in their presentations to students, or as documents for them to study or work on (e.g.: books scanned by them and sent to their students, an extract of a song or movie showed in a class). Is it necessary, in these cases, to request the consent of copyright holders? Is it necessary for the teacher or professor to contact the author of the work for authorizing its use in classes?

The pandemic caused by COVID-19 has not meant a relaxation of copyright or their repeal. These rights have been fully in force. The copyright in the online environment is regulated in two European directives: Directive 2001/29/EC of the European Parliament and of the Council of 22 May 2001 on the harmonisation of certain aspects of copyright and related rights in the information society, and Directive (EU) 2019/790 of the European Parliament and of the Council of 17 April 2019 on copyright and related rights in the Digital Single Market and amending Directives 96/9/EC and 2001/29/EC. As a general rule, we can say that authors and other copyright holders (such as singers, actors or producers) have two exclusive rights that they can exercise in the digital networks’ context: the right of reproduction and the right of communication to the public. According to the first, only their holders can authorize or prohibit the making of copies of their works or subject matters. It does not matter if the reproduction is direct or indirect, temporary or permanent, in whole or in part (article 2 of Directive 2001/29). According to the right of communication to the public, only their holders can authorize or prohibit the making available to the public of works or subject matters, by wire or wireless means, in such a way that members of the public may access them from a place and at a time individually chosen by them (article 3 of Directive 2001/29).

However, the European legislator, from some years ago, has planned some limitations or exceptions to copyright for teaching purposes. They seek to achieve a fair balance between the rights and interests of authors and other rightholders, on the one hand, and of users on the other. Some of these limitations were already provided for in the Berne Convention for Protection of Literary and Artistic Works (1886), since its revision at the Rome Conference in 1928, in a very ambiguous and broad way.^1^ Therefore, teachers and professors can use copyrighted content without having to ask for the authorization of copyright holders because the legislator authorizes it. However, it does not mean that works and subject matters can be used in any way, because there are some requirements that European regulations state. And if we do not follow those requirements, we will infringe copyright.

The three limitations that have to do with online teaching are private copying, quotation and illustration for teaching. All they are regulated in Directive 2001/29, and Directive 2019/790 regulates a specific limitation for teaching when it is developed in a cross-border context. The problem is that these three limitations included in Directive 2001/29 are optional for Member States and it leaves them flexibility in the way to implement them in their national law. Nevertheless, the Directive 2001/29 does not allow them to include more extensive limitations than those set out in its article 5 (recital 32 of Directive 2001/29). Member States have to respect all the individual requirements provided for the limitations. Any broader condition is not allowed, although they may introduce more restrictive requirements or stronger conditions in every limitation. This makes impossible to reach the goal of harmonization of limitations mentioned in recital 31 of Directive 2001/29 and this creates a multiplicity of regulations and complicates the use of works in remote teaching activities.^2^ In contrast, the limitation of illustration for teaching in a cross-border context, provided for in article 5 of Directive 2019/790, is mandatory for Member States. According to its recital 5, the optional nature of exceptions provided for in Directive 2001/29 could negatively impact the functioning of the internal market. The European legislator is aware of the importance of digital technologies in the field of teaching and education, in particular as regards cross-border uses. Therefore, a mandatory exception of illustration for teaching must be introduced in the national law of Member States, just to allow those new uses. The purpose of all of them is to encourage the dissemination of knowledge.^3^

On the other hand, when applying these limitations, we have to take into account the so-called “three-step test”, also included in article 9(2) of Berne Convention and in article 5(5) of Directive 2001/29. It means that limitations shall be applied only in certain special cases that do not conflict with the normal exploitation of the works or other subject matter and do not unreasonably prejudice the legitimate interests of the copyright holders. Therefore, we have to make a restrictive interpretation when applying a limitation or exception to copyright.

## Wrong ideas about using contents in teaching

Before analyzing the limitations, it is necessary to start from two wrong ideas that teachers and professors normally have in mind. The first one: if the content is freely accessible on the Internet, we can do whatever we want with it. However, free access does not mean a free use too, as the material may be protected by copyright. Therefore, if we find an image in Google Images, for example, we will have to find out if it is protected by copyright or if a free use is allowed by a Creative Commons license.^4^ But do not panic! If it is protected by copyright, teachers and professors can use it according to one of the copyright limitations, but not in such a wide way as in the cases of Creative Commons licenses.

The second idea: if we are the authors of a work, we can share it with our students without any restriction. That is not true. We must separate the condition of author from the condition of copyright holder. The condition of author is not transferable and we will always have it in our hands. But the fact that we are the authors of a certain work does not mean that we can share it freely, since it may happen that we have transferred our exploitation rights to a third party (e.g.: a publisher). If that is the case, we have to ask for permission from that third party to use the work, despite being the authors. For example, if I wanted to share this entire article with my students, I should ask Springer, as publisher, for permission, because it has my exploitations rights on this work as I transferred them to it for the publication. All this without prejudice to the possibility of applying any copyright limitation, as it is going to be explained right now.

## Private copy exception

The limitation on private copying is included in article 5(2)(b) of Directive 2001/29. The reason why the legislator allows private copies is the impossibility of their control and the right to privacy of users of works and subject matters. According to this limitation, a teacher or professor could make a copy of a whole work (e.g.: a book) to prepare a lesson. The making of private copies requires to observe the following conditions:

1. The copy must be made by a natural person. Legal persons cannot make private copies. So, we have to make the copy by ourselves (e.g.: with my own photocopier or scanner). The copies that are made in reprographic establishments are not private copies, but authorized copies by the copyright holder.^5^
2. The copy must be destinated to the private use of the copyist. Private use implies that only the copyist (the teacher or professor) can use the copy. Instead, we cannot share the copy with an indeterminate number of people, such as the students in a class. Also, we have to keep in mind that the only right that is limited by this exception is the right of reproduction, not the right of communication to the public. If we share the copies with our students, we will be affecting that former right.
3. Commercial purposes are not allowed. The teacher or professor, therefore, could not make copies of a book and sell them to the students.
4. The source from which a reproduction for private use is made must be lawful. The Court of Justice of the European Union (CJEU) has reached this conclusion in its Judgment of 10 April 2014 (*ACI Adam BV and Others v. Stichting de Thuiskopie, Stichting Onderhandelingen Thuiskopie vergoeding*, Case C-435/12). In its opinion, the making of private copies from unlawful sources is not included in the exception due to the following reasons: (1) it could clearly be detrimental to the proper functioning of the internal market of the European Union; (2) the objective of proper support for the dissemination of culture stated in recital 22 of Directive 2001/29 must not be achieved by sacrificing strict protection of rights or by tolerating illegal forms of distribution of pirated works; (3) that kind of copies may infringe certain conditions laid down by article 5(5) of Directive 2001/29, because such reproductions made from unlawful sources would encourage the circulation of pirated works, reducing the volume of sales or of other lawful transactions relating to protected works and affecting the normal exploitation of those works and the interests of copyright holders.

On other hand, for the application of this limitation is not necessary to be the owner of the work to be copied, but it is perfectly possible to borrow it from a library and make a private copy or to access the work through a library database and save a copy of it on a memory stick. Private copy limitation may be extended to acts such as the storage of works on a USB stick, carried out by users from dedicated terminals installed in publicly accessible libraries according to article 5(3)(n) of Directive 2001/29, provided that all the conditions laid down by those provisions are met. That is what the CJEU said in its Judgment of 11 September 2014 (*Technische Universität Darmstadt v. Eugen Ulmer KG*, Case C-117/13). Finally, it can be copied any kind of work: literary, musical or audiovisual.

## Quotation limitation

The limitation about quotation is included in article 5(3)(d) of Directive 2001/29. The European legislator has allowed this limitation because of the freedom of speech and the freedom of opinion. Quoting implies including extracts of someone else's work in one's own work (e.g.: we reproduce some extracts of someone else’s work in the material we are preparing for our students to support an opinion). In order to do so, the following requirements must be met:

1. We have to use works for review or critical purposes. Aesthetic or beautification purposes are not allowed. Furthermore, the quotation must be related to the issue we are dealing with in our work.
2. We can only use works or subject matters that have been previously made legally available to the public. A work will be legally made available to the public when copyright holder has authorized it. We cannot quote unpublished works or subject matters. According to the CJEU, a work has already been lawfully made available to the public where that work, in its specific form, was previously made available to the public with the rightholder’s authorization or in accordance with a non-contractual license or statutory authorization ((Judgment of 29 July 2019, *Spiegel Online GmbH v. Volker Beck*, Case C-516/17). Anyway, it is possible to quote any kind of work (literary, musical or audiovisual). Even a lecture could be quoted because it would meet this requirement.
3. The source must be indicated, including the name of the author, except in cases where it is impossible. For example, it could be impossible in relation to anonymous works, or in relation to works published under a pseudonym, or when there are many authors in a work and it is not possible to distinguish which part corresponds to each one. The aim of this requirement is to avoid that the people confuse the authorship of everything included in a work. If we do not indicate the name of the author, we could commit a plagiarism and we could infringe the moral right of attribution of author. In addition, it also serves to increase the fame of the author, because the more mentioned he/she is, the more authority he/she will have in that field. These indications will let the readers go to the quoted work in the original version. The teacher or professor has to develop a minimal degree of care in searching for the name of the author.^6^ In any case, Directive 2001/29 does not give any guidelines on how such indications should be made.
4. It is necessary to make a fair practice. We have to use only the extracts that are necessary for our purposes. We cannot turn our own work into a collection of quotations. Our work must maintain originality and preserve its structure if we remove the quotations of other people's works. Moreover, the extracts must occupy an accessory place in our work. In addition, the public must be fully aware that this excerpt corresponds to someone else's work (e.g.: when we use italics, we are explaining that the extract belongs to another author). The CJEU has indicated that quotation limitation requires that the quoted work can be clearly identified in the work that includes it (Judgment of 29 July 2019, *Pelham GmbH, Moses Pelham, Martin Haas v. Ralf Hütter, Florian Schneider-Esleben*, Case C-476/17).
5. In terms of length, we must only use what the specific purpose proposed requires. It is not necessary to reproduce the whole work for criticism or review purposes, because the use of an extract could already be enough. The problem is that the European legislator has not defined what an excerpt is (how many sentences can we copy from a work for quotation?). When a work is long, it may be understood that a paragraph of two or three sentences is an extract. However, when we want to quote short works (e.g.: a haiku), it is more difficult to determine it. In those cases, it is possible to quote the whole work, because to quote only an excerpt could give a wrong idea about the work.^7^ In any case, the length of quotation will have to be determined in relation to the total length of the work that we are going to quote. For example, if the work that is going to be quoted is two pages long, maybe to reproduce half a page is excessive. Nonetheless, if instead of reproducing excerpts we use a link that gives access to the entire work, there would be no violation of copyright. The CJEU has stated that the concept of quotation of article 5(3)(d) of Directive 2001/29 covers a reference made by means of a hyperlink to a file which can be downloaded independently (Judgment of 29 July 2019, *Spiegel Online GmbH v. Volker Beck*, Case C-516/17). In those cases, we have to take the requirements about linking into account.^8^

Thus, a teacher or professor could prepare a PowerPoint presentation including extracts of literary works obtained from other people's works, to support the ideas. As CJEU said, it is irrelevant if the quotation is made as a part of a work protected by copyright or as part of a subject matter not protected by copyright (Judgment 1 December 2011, *Eva-Maria Painer v. Standard VerlagsGmbH, Axel Springer AG, Süddeutsche Zeitung GmbH, Spiegel-Verlag Rudolf Augstein GmbH & Co KG, Verlag M. DuMont Schauberg Expedition der Kölnischen Zeitung GmbH & Co KG*, Case C-145/10). It is not necessary to reproduce the extracts literally, because it is possible to paraphrase, but if they are not copied literally, we must be especially careful not to distort the thinking of the author. However, the translation of the extract to be quoted is not allowed. The limitation allows us only to reproduce and to communicate to the public, but not to translate into another language, unless we include the original version.

## The exception about illustration for teaching

The limitation on illustration for teaching is established in article 5(3)(a) of Directive 2001/29. According to this limitation, teachers and professors will be able to reproduce and communicate to the public works and subject matters among their students, for educational purposes (e.g.: to photocopy two pages of a book and send them to the students). We have to fulfill the following requirements:

1. Only teaching purposes are allowed. The work must be used only to illustrate what is being taught. Any other purpose will not be covered by article 5(3)(a) of Directive 2001/29. The use of the work does not have to be made during teaching, but it can be used as a material for students.^9^ This limitation includes the use of works to explain a topic or to be read and commented by our students, for example. Copyrighted content could also be used for the purpose of evaluating students through exams, since evaluation purposes are also covered by the teaching purpose. So, we could include an extract of a book to be commented in an exam, for example. On the other hand, it is not necessary for the teacher or professor to make the copy on him/her own, but the reproduction can be made by a third party. Moreover, we can send the work or subject matter to the students by any kind of platform, including an intranet, email or WhatsApp. It is very important that only our students have access to such material (we cannot give access to it to anyone in the public).
  In addition, the exception covers the teaching purpose at any level, namely schools, high schools or universities. No matter whether these institutions are public or private.^10^ The most important is that teaching and learning activities are carried out under the responsibility of educational institutions, although it is distance instruction. Therefore, the limitation does not allow the use of works in teaching through a broadcasted TV-show.
2. We can use any kind of work: literary, musical, audiovisual, photography… However, certain works cannot be used just not to infringe the three-step test. For example, it would not be possible to use a university textbook, because it could conflict with the normal exploitation of that kind of work. If the teacher or professor scans several pages of a Civil Law textbook, the students will not buy it, taking away income from its author. Instead of that, it would be possible to do the reading of an extract from a university textbook or its screening, provided that the students do not have access to it.
3. The source must be indicated, including the name of the author, unless this turns out to be impossible. It is the same requirement as for quotation.
4. We can only use works or subject matters that have been previously made legally available to the public.
5. The limitation allows us only to reproduce and to communicate to the public the copyrighted material, but not to translate it into another language, unless we include the original version.
6. We must only use works to the extent that it is justified by the educational purpose pursued. This limitation should not allow the use of the whole work to illustrate a class (e.g.: playing an entire movie for our students in the class), as this would affect the normal exploitation of the work and would harm the legitimate interests of copyright holders. We could only use excerpts of literary, musical or audiovisual works in our classes, unless it is a short work (e.g.: a poem) or a photograph, where it is possible to use the whole.^11^ Thus, we could send to our students or upload on a platform to which only they have access, extracts of books, songs, movies or TV-shows, as long as they are related to what is being explained in class.
  We have the same problem as in the quotation limitation, because the European legislator does not specify how long the extracts should be. In Spain there are two limitations related to the illustration for teaching. The first one allows the free use of extracts from literary, musical, and audiovisual works. Likewise, it allows the use of entire works of fine art and photographic works. This first limitation is intended for teaching at any level. The second of the limitations is established only for university professors and it allows only the use of literary works. Under this exception, a professor may reproduce, communicate to the public and distribute a book chapter or a journal’s article, provided that the 10% of the total length of the work is not exceeded. In this case, the university must pay remuneration to the rights holders through a collective management organization.
  On the other hand, we could not show an entire YouTube video in a class, despite it is freely accessible in that platform. We can only use extracts of those videos. The use of entire YouTube videos would affect its business model of advertising revenue calculated based on the number of visits. If the teacher or professor shows a YouTube video in a class with 30 students, for example, he/she only clicks once on the video and not 30, affecting the revenue YouTube receives from advertising. In these cases, if we want students to watch the entire video, we have to indicate the link for each student to click on the video and do it from their device.
  The CJEU has indicated several times that the provision on a website of clickable links to works or subject matters does not constitute an act of communication to the public that requires authorization (Judgment 13 February 2014, *Nils Svensson, Sten Sjögren, Madelaine Sahlman, Pia Gadd v. Retriever Sverige AB*, Case C-466/12; Order 21 October 2014, *BestWater International GmbH v. Michael Mebes, Stefan Potsch*, Case C-348/13; Judgment 8 September 2016, *GS Media BV v. Sanoma Media Netherlands BV, Playboy Enterprises International Inc., Britt Geertruida Dekker*, Case C-160/15). We can use all types of links: a simple link, a deep link or even a frame.^12^ However, the CJEU has stated that two requirements must be met. Firstly, the work in question must be already available with unrestricted access on the website to which the hyperlink provides access. The access is restricted when there is a measure to control the access to the work in anyway or when we have to pay or subscribe for accessing to that linked work.
  Secondly, the linked work or subject matter must have been made available to the public on the linked website with the consent of copyright holders. It is not necessary for the teacher or professor to carry out an investigation on whether the work to which he/she wants to link was uploaded with the authorization of copyright holders or not. As this is very difficult to determine, the CJEU has pointed out the following presumption: when the posting of a hyperlink to a work freely available on another website is carried out by a person who, in so doing, does not pursue a profit, it is accordingly necessary to take account of the fact that that person does not know and cannot reasonably know, that that work had been published on the internet without the consent of the copyright holder. Furthermore, when the posting of hyperlinks is carried out for profit, it can be expected that the person who posted such a link carries out the necessary checks to ensure that the work concerned is not illegally published on the website to which those hyperlinks lead, so that it must be presumed that that posting has occurred with the full knowledge of the protected nature of that work and the possible lack of consent to publication on the internet by the copyright holder. The teachers and professors will be non-profit making and, in many cases, the aspect of the website where this linked content is uploaded can already give us clues about the existence or not of such authorization. Therefore, it is not possible to link to content whose access is restricted or to content that has not been made available to the public in an authorized way (e.g.: linking to piracy copies).
7. Commercial purposes are prohibited. When applying this limitation, the non-commercial nature of the activity in question should be determined by that activity as such. Thus, this limitation does not allow: (1) the teacher or professor to charge the students an amount of money to have access to the material; (2) the use of copyrighted content by a private teacher in relation to his/her teaching activities developed outside an official educational institution (e.g.: a teaching learning center). All of them would have commercial purposes. However, this requirement does not mean that the teacher or professor cannot earn a specific fee for the class where he/she has used the copyrighted material, provided that teaching is developed in an official educational institution (e.g.: a class in a Master’s degree). Furthermore, according to recital 42 of Directive 2001/29, the organizational structure and the means of funding of the establishment concerned are not the decisive factors in this respect. So, it is possible to apply this limitation to public institutions and to private institutions, as it was said before.

When teaching is developed in cross-border courses, article 5 of Directive 2019/790 states how to proceed. Cross-border teaching takes place at a distance (remote teaching), beyond the borders of the Member State where the educational center is located. According to recital 19, it is necessary to ensure that educational establishments benefit from full legal certainty when using works or other subject matter in digital teaching activities, including online and across borders. In these cases, the teacher or professor will also be able to use copyrighted material with their students to illustrate their explanations. In this case, the requirements are the following:

1. The use must be justified by the teaching purpose. The limitation provided for in article 5 of Directive 2019/790 benefits all educational establishments recognized by a Member State, including those involved in primary, secondary, vocational and higher education (recital 20 of Directive 2019/790). The beneficiary institutions and the recipients of the works are defined in a very broad way, but this limitation excludes teaching uses made by other kind of institutions, such as libraries or museums.^13^ It covers digital uses of works or other subject matter to support, enrich or complement the teaching, including learning activities and during examinations or teaching activities that take place outside the premises of educational establishments, for example in a museum, library or another cultural heritage institution. The limitation should cover uses of works or other subject matter made through digital means, for example electronic whiteboards or digital devices which might be connected to the internet.
2. It is not possible to use the whole work or subject matter. The limitation implies the use only of parts or extracts of works, which should not substitute for the purchase of materials primarily intended for the educational market. Furthermore, recital 21 of Directive 2019/790 also clarifies that uses allowed under the exception or limitation should be understood to cover the specific accessibility needs of persons with a disability in the context of illustration for teaching.
3. Commercial purposes are not allowed. This requirement must be understood as analyzed in relation to the exception of article 5(3)(a) of Directive 2001/29. Moreover, the organizational structure and the means of funding of an educational establishment should not be the decisive factors in determining whether the activity is non-commercial in nature (recital 20 of Directive 2019/790).
4. To avoid the dangers of online communication, the use has to be made through a secure electronic environment that can only be accessed by the students and the teaching staff of the center. Therefore, closed and internal networks of the educational center are required. Secure electronic environments should be understood as digital teaching and learning environments access to which is limited to an educational establishment's teaching staff and to pupils or students enrolled in a study programme, in particular through appropriate authentication procedures including password-based authentication (recital 22 of Directive 2019/790).
5. The source must be indicated, including the name of the author, unless this turns out to be impossible.

In these cases, Member States may provide that this limitation does not apply as regards specific uses or types of works or subject matters, such as material that is primarily intended for the educational market, workbooks or scores, to the extent that suitable licenses authorizing the acts referred before and covering the needs and specificities of educational establishments are easily available on the market.^14^ Thus, university textbooks could not be scanned to make them freely available to students because that would affect their normal exploitation and would take away income from copyright holders. In relation to another limitation included in Directive 2001/29, the CJEU has stated that the concept of “purchase or licensing terms” must be understood as requiring that the copyright holder and an establishment must have concluded a licensing agreement in respect of the work in question that sets out the conditions in which that establishment may use that work (Judgment 11 September 2014, *Technische Universität Darmstadt v. Eugen Ulmer KG*, Case C-117/13). However, the European legislator does not require a concluded licensing agreement, but an available license. This should incentivize licensing solutions.^15^ But, if an institution has not acquired a license for the use of that kind of material, then the teacher or professor will have to ask for the authorization of copyright holder on him/her own (e.g.: asking for it to a collective management organization).^16^ On the other hand, this mechanism of licensing does not mean necessarily that they will be low-cost or affordable.^17^

Finally, Member States may provide for fair compensation for copyright holders for the use of their works or other subject matter according to the limitation of article 5 of Directive 2019/790.

## Conclusion

In conclusion, the pandemic situation has forced us to make an effort to continue with our teaching work. However, intellectual property rights must be observed and respected. The European legislator looks after the interests of teachers/professors and students, allowing the use of works and subject matters for educational purposes without having to ask for permission from the copyright holders. Nonetheless, it is very important to meet all the requirements included in the European directives just to do not infringe copyright.

Teachers and professors can use all kinds of works (literary, musical and audiovisual) for the development of their classes. Among other things, they will:

- Make a private copy of a work to prepare their classes.
- Include fragments of works in their Powerpoints presentations or in the material that they create, to make a study, criticism or comment of that work.
- Scan excerpts of literary works to send them to their students or upload them to course platforms, for them to study, work on or simply as a complement to the explanations.
- Use excerpts of musical works or audiovisual works to complement the explanations or to be worked on by their students.
- Include extracts of works in the exams for the evaluation of the students.
- Provide links to copyrighted content so that students can access it.

Copyright does not constitute an obstacle to the use of works in the educational field. Teachers and professors will be able to carry out their work in the online environment with the peace of mind that they can use all this content without infringe copyright.

## Footnotes

1 For a study of these limitations in Berne Convention, see Gervais (2008, pp. 3–41) and Xue (2008, pp. 213–229).

2 Trimble (2020, p. 407).

3 Latreille (2020, p. 210) and Aboyeji (2020, p. 45).

4 The Creative Commons licenses are licenses granted by authors so that the general public can use their works freely and for free, although they may sometimes impose some restrictions (e.g.: the prohibition of transformation or the prohibition of lucrative use of the work). These licenses are tools to get over the restrictions set out in copyright laws and the traditional “all rights reserved” clauses. The works subject to a Creative Commons license are easily noticeable, because the author uses a set of symbols. For further information: https://creativecommons.org/licenses/?lang=en.

5 Walter and Von Lewinski (2010, p. 1033).

6 Walter and Von Lewinski (2010, p. 1044).

7 Gaubiac (1997, p. 13) and Walter and Von Lewinski (2010, p. 1050).

8 The requirements about linking will be explained in Sect. 5.

9 Bechtold (2006, p. 378).

10 Walter and Von Lewinski (2010, p. 1043).

11 Walter and Von Lewinski (2010, p. 1043).

12 A simple link contains only the URL address of the site to which it links. After the link has been clicked on, that home page is opened either in place of the page containing the link or in a new window. The address bar of the browser displays the URL address of the new site, so that the user is aware that he or she has changed sites. A deep link leads not to the home page of the target site, but to another page on that site or even a specific resource contained on that page (e.g.: a photography or text. Each page and each resource have a URL address which can be used in the link instead of just the main address of the site. A deep link circumvents the home page of the website. However, since the URL address of a webpage generally contains the name of the site, the user is always informed of the site which he or she is viewing. Framing is a technique which allows an external resource, such as a website, a webpage or an individual element of a website (e.g.: a video), to be placed in a frame inside the website of the person who provide the kind of link. The frame is a means of embedding external resources in a webpage.

13 Quintais (2020, p. 32).

14 This provision should be welcomed because it tries to accommodate the limitation to the three-step test. However, the use of such works should be allowed when it does not involve making them available to students (e.g.: the professor reading an extract of a university textbook in the classroom). Some authors think that this provision neglects the public interest dimension underpinning of the limitation. See Quintais (2020, p. 32).

15 Shapiro (2016, pp. 12–772).

16 Jütte (2019, p. 671).

17 Latreille (2020, p. 220).
